# The Mediation Effect of Peripheral Biomarkers of Calcium Metabolism and Chronotypes in Bipolar Disorder Psychopathology

**DOI:** 10.3390/metabo12090827

**Published:** 2022-09-02

**Authors:** Renato de Filippis, Martina D’Angelo, Elvira Anna Carbone, Pasquale De Fazio, Luca Steardo

**Affiliations:** 1 Psychiatry Unit, Department of Health Sciences, University Magna Graecia of Catanzaro, 88100 Catanzaro, Italy; 2 Psychiatry Unit, Department of Medical and Surgical Sciences, University Magna Graecia of Catanzaro, 88100 Catanzaro, Italy

**Keywords:** bipolar disorder, calcium metabolism, chronobiology, chronotype, circadian rhythm, parathormon, sleep, vitamin D

## Abstract

Calcium (Ca^++^) metabolism may be impaired in several psychiatric diseases. We hypothesize that calcium imbalance might also correlate with a specific chronotype and could be recognized as a marker of illness severity in bipolar disorder (BD). We aimed to (1) identify the association between calcium imbalance and a specific chronotype in a cohort of BD patients, and (2) test the mediation role of high parathyroid hormone (PTH) levels towards a specific chronotype and illness severity in BD patients. Patients’ socio-demographic and clinical characteristics were collected with an ad-hoc schedule. We administered the Hamilton Depression Rating Scale (HAM-D), the Hamilton Rating Scale for Anxiety (HAM-A), the Young Mania Rating Scale (YMRS), and the Morningness Eveningness Questionnaire (MEQ). 100 patients affected by BD were recruited. The Kruskal-Wallis test showed a significant difference between the three MEQ groups in PTH levels (*p* < 0.001) and vitamin D levels (*p* = 0.048) but not in Ca^++^ levels (*p* = 0.426). Dwass-Steel-Critchlow-Fligner Pairwise analyses performed concerning three MEQ groups revealed significantly higher scores on PTH levels in MEQ-E subjects compared to MEQ-M and MEQ-I (in both cases, *p* < 0.001). No differences emerged between calcium levels among the three chronotypes. The mediation analysis has shown that elevated PTH levels are directly influenced by more severe HAM-A, HAM-D, and YMRS scores. MEQ-E could be a marker related to BD and predispose to various factors influencing mood symptoms. The combination of vitamin D therapy in MEQ-E may help to improve prognosis in this subtype of patients affected by BD.

## 1. Introduction

The role of calcium metabolism in psychiatric disorders recently received increasing research focus. Three main research directions support the need to implement studies investigating the role of parathyroid hormone (PTH), vitamin D, and serum calcium (Ca^++^) in mental health conditions [1], particularly in mood disorders [2,3,4,5,6]. Firstly, vitamin D regulates both genomic and non-genomic activities. In particular, genomic functions are mediated through the interaction of the most active form of vitamin D (i.e., 1 α,25(OH)_2_ vitamin D) with the vitamin D receptors (VDRs), which is a DNA-binding transcription factor that regulates the expression of genes contained in their promotor region, such as the vitamin D response element (VDRE) [7]. There is an increased expression of VDRs in specific brain regions involved in mood regulation, such as the prefrontal cortex and the limbic system [8,9]. Secondly, it has been demonstrated a putative regulatory role, exploited by vitamin D, in the association between depression and neuroinflammation, through its immune-modulatory properties [10]. Thirdly, Vitamin D can regulate the production of specific neurotrophins (i.e., nerve growth factor, glial cell-line derived neurotrophic factor, etc.) and displays a neuroprotective function due to its anti-inflammatory effect. Vitamin D also underlies the human brain’s regulatory function by synthesizing many neurotransmitters, including serotonin, which can influence mood levels and fluctuations in several psychiatric disorders [11]. Finally, genetic studies have associated gene alterations related to calcium, circadian rhythm, cell growth/development, and brain connectivity with mood disorders, including bipolar disorders [12]. Experimental evidence has shown that Ca^++^ signaling plays a crucial role in regulating molecular rhythms of circadian locomotor output cycles (CLOCK) genes and circadian behavior. Ca^++^ is fundamental to synchronizing the neuronal networks that make up circadian pacemakers [13]. In turn, the activity of circadian clocks may influence Ca^++^ signaling. For instance, several genes that encode Ca^++^ channels and Ca^++^-binding proteins exhibit rhythmic expression, so a disruption of this cycling affects circadian function, reinforcing the notion of an important reciprocal relationship between them [14].

Interestingly, several genes coding for proteins associated with Ca^++^ pathways exhibits rhythmic transcription within various biological clocks [15]. Moreover, PTH can also be considered an accurate marker of chronic calcium homeostasis imbalance [16]. PTH regulates circulating and intracellular calcium levels in the central nervous system (CNS), inducing apoptosis due to calcium overloading [17]. Elevated PTH levels are associated with a reduced regional cerebral blood flow, whereas PTH-related protein (PTHrP) inhibits the activity of the calcium channel, thus contributing to neuronal homeostasis [18]. Secondly, chronic high levels of PTH could be associated with neural damage. Instead, a higher calcium concentration can be found in the CNS because of PTH imbalance. Therefore, such experimental evidence led clinicians to thoroughly investigate the effect of calcium metabolism imbalance on psychiatric disorders. Our previous study demonstrated a strong correlation between calcium imbalance and a higher psychopathological burden in bipolar disorder (BD) [19]. Those results were explained considering a possible correlation between the presence of high multiple traumatic experiences capable of triggering a neuroinflammation response and, consequently, inducing abnormal changes in several physiological processes. In mammals, circadian clocks modulate the daily rhythmicity of most physiological processes [20]. At the cellular level, circadian rhythms are managed by intracellular transcriptional/translational feedback loops (TTFL) [21]. Increasing evidence demonstrates that they can also be regulated by multiple signaling pathways, including Ca^++^ signaling, which acts as a crucial factor in setting the molecular rhythms of CLOCK genes and the resulting circadian behavior. Moreover, in vivo imaging studies have proven that Ca^++^ is crucial in synchronizing the neuronal networks that constitute circadian pacemakers. On the other hand, the activity of circadian clocks can, in turn, affect Ca^++^ signaling. For example, several genes encoding for Ca^++^ channels and Ca^++^-binding proteins exhibit a rhythmic expression, and the breaking of this cycling impacts circadian function, reinforcing the notion of their reciprocal link [22].

Finally, when referring to chronotype preference or morningness–eveningness, we mean the individual preference of the time of day for carrying out primary working, social, and/or personal activities [23], and, therefore, it usually reflects the 24 h or ultradian propensity for the individual either to be alert or to sleep. In this regard, three different chronotypes are usually identified, i.e., morning types, evening types, and neither (indifferent) [24].

Thus, chronotypes and circadian rhythmicity are closely related, and they could be studied together. Indeed, circadian rhythmicity describes variability, stability, and period functional performance. Biological, clinical, and psychopathological measures may provide elements of circadian rhythmicity [25].

Within this context, one of the primary purposes of the current study was to investigate the association between calcium imbalance and a specific chronotype in a sample of BD. Specifically, the current study was designed to explore whether calcium metabolism imbalance could directly affect circadian rhythmicity and worsen BD outcomes. Indeed, we hypothesize that calcium imbalance might correlate with a specific chronotype and could be recognized as a marker of severity of illness in BD. Particularly, in a previous study, we showed that high levels of PTH are correlated to the worst outcome and high psychopathological burden in BD. Therefore, the aims of the present study are:

(1) identifying the association between calcium imbalance and a specific chronotype in a cohort of BD patients;
(2) testing the mediation role of high PTH levels between a specific chronotype and illness severity in BD patients.

## 2. Material and Method

Patients were consecutively recruited at the Psychiatric Units of the University Hospital Mater Domini in Catanzaro from June 2019 to March 2020. Patients were included in the study if they met the following criteria: (1) age between 18 and 65 years; (2) diagnosis of type-I (BD-I) or type-II (BD-II) BD, according to the Diagnostic and Statistical Manual of Mental Disorders—fifth edition (DSM-5) [26]; and (3) willingness to participate in the study. Patients were excluded in case of (1) an inability to provide written informed consent to participate in the study, (2) moderate or severe cognitive impairment, as assessed by Mini Mental State Evaluation (MMSE) <22, (3) comorbidity with any neurologic disease or substance and/or alcohol use disorder, (4) being pregnant or in the post-partum period, and (5) currently in treatment with medications that could alter the calcium metabolism such as vitamin D supplementation or calcium phosphonate. All patients gave their written informed consent to participate in the study after a complete description of its aims and design. The study was carried out following the latest version of the Declaration of Helsinki and was approved by the Ethics Committee of the University of Catanzaro (307/2020). 

### 2.1. Procedures and Measures

Patients’ socio-demographic and clinical characteristics, including sex, age at study entry, employment status, educational level, family history of psychiatric illnesses, type of onset, lifetime number of affective episodes, the pattern of illness course, history of suicide attempts, antidepressant switch, psychotic features, aggressive behavior, mixed features, anxious features, age of first contact, and age at onset were collected with an *ad-hoc* schedule. The BD clinical severity was assessed by Hamilton Depression Rating Scale (HAM-D) [27], the Hamilton Rating Scale for Anxiety (HAM-A) [28], and the Young Mania Rating Scale (YMRS) [29]. Chronotype was evaluated using the Morningness Eveningness Questionnaire (MEQ) [30], a self-administered scale assessing sleep-wake rhythms and circadian preference concerning specific activities. The MEQ is a 19-item scale with a total score range from 16 to 86. Scores up to 41 underpin an “evening type”, scores between 42–58 indicate an “intermediate type”, and scores of ≥59 point towards a “morning type”. Biological samples were obtained from all patients to determine the serum levels of calcium (mmol/L), 25-OH-vitamin D (ng/mL) and PTH (pg/mL). They were assessed at recruitment in the laboratories of the two participating sites, adopting a standardized procedure. Calcium was measured using standard laboratory methods. Blood was centrifuged, and serum was stocked at −30 °C for 1 α,25(OH)_2_ vitamin D and PTH and evaluated by chemiluminescence immunoassays using adequate kits (Diasorin Liaison; ADVIA Centaur).

### 2.2. Statistical Analysis

Descriptive statistics were calculated for socio-demographic and clinical characteristics and other relevant assessment instruments. Where appropriate, data are presented as means and standard deviations (SD) or frequencies and percentages (%). The Shapiro-Wilk test was adopted to check the normality of distribution of continuous variables, such as age, age at illness onset, age of first psychiatric consultation, and the total number of affective episodes in our sample. The PTH, calcium, and vitamin D were compared across chronotypes groups defined by qualitative variables through nonparametric Kruskal-Wallis’s tests. Differences between socio-demographic and clinical variables across three different chronotypes were measured using the χ^2^ test. The Dwass-Steel-Critchlow-Fligner pairwise comparisons were performed to test the differences between the chronotypes and the total score of psychometric scales (HAM-D, HAM-A, and YMRS) and biological variables (Ca^++^, PTH, and vitamin D).

Following preliminary analyses carried out on the entire sample, further sub-analyses (U Mann-Whitney’s test, χ^2^ Test) were run considering the sample divided into the MEQ-E^+^ group (subjects with an eveningness chronotype) and the MEQ-E^-^ group (subjects without an eveningness chronotype). Given that, an association emerged in MEQ-E^+^ subjects between psychopathological and PTH levels, and the Sobel test was run to investigate the effect of PTH as a mediator factor between MEQ-E and a more severe psychopathological burden in BD. Furthermore, a mediation analysis was performed using the Jamovi program [31] to test the effect of PTH levels between MEQ-E and all clinical variables statistically significant to previous analyses and indicated as predictive of a greater psychopathological load. The level of statistical significance was set at a nominal value of *p* ≤ 0.05. Statistical analyses were performed using SPSS 26 version [32].

## 3. Results

The total sample included 100 subjects affected by BD (55 BD-I and 45 BD-II), 50% males, with a mean age of 46.5 ± 13.9 years. Almost half of the patients were in a stable relationship, were employed, and had a positive history of psychiatric disorders. The mean age at onset was 27.3 ± 9.9, and the duration of illness was 19 ± 12.99 years. Thirty-two subjects were classified as having an eveningness chronotype (MEQ-E), 41 with an intermediate chronotype (MEQ-I), and 27 with a morningness chronotype (MEQ-M). These three chronotype groups were homogeneous for age (*p* = 0.978), sex (*p* = 0.058), age at illness onset (*p* = 0.716) and age at first psychiatric consultation (*p* = 0.886) (Table 1).

Significant differences emerge from the bivariate analyses between the three chronotype subgroups (Table 2 and Table 3).

A higher number of lifetime depressive episodes and lifetime total affective episodes were expressed in MEQ-E subjects compared to MEQ-M and MEQ-I (in both cases and both chronotypes, *p* < 0.001) (Table 3). A higher number of hypomanic episodes were reported in MEQ-E+ compared to MEQ-E- (*p* = 0.018). MEQ-E+ subjects more significantly reported antidepressant switch (*p* < 0.001), mixed features (*p* < 0.001), psychotic features (*p* < 0.001) and history of suicide (*p* < 0.001), compared to MEQ-E- subjects (Table 2). The Kruskal-Wallis test across three chronotypes showed differences in total scores at HAM-D (*p* < 0.001), HAM-A, and YMRS (*p* < 0.001) between MEQ-E, MEQ-I, and MEQ-M. Nonetheless, a significant difference was found across the three MEQ groups in PTH levels (*p* < 0.001) and in vitamin D levels (*p* = 0.048), but not in Ca^++^ levels (*p* = 0.426) (Table 3). The Dwass-Steel-Critchlow-Fligner Pairwise analyses carried out concerning three MEQ groups revealed significantly higher scores at PTH levels in MEQ-E subjects compared to MEQ-M and MEQ-I (in both cases, *p* < 0.001), with only a borderline significance at vitamin D levels between MEQ-E and MEQ-M (*p* = 0.047). However, when we classified the sample in MEQ-E+ and MEQ-E-, the Mann-Whitney U test revealed significantly higher vitamin D levels in MEQ-E- (*p* = 0.022). Similarly, pairwise comparisons found significantly higher HAM-A total scores in MEQ-E subjects compared to MEQ-I (*p* < 0.001) and MEQ-M (*p* < 0.001), but no differences between MEQ-I and MEQ-M (*p* = 0.826). In addition, the Dwass-Steel-Critchlow-Fligner pairwise analysis identified a difference in HAM-D total scores between MEQ-E and MEQ-I (*p* < 0.001) as well between MEQ-E and MEQ-M (*p* < 0.001), but no difference was detected between MEQ-I and MEQ-M (*p* = 0.925). Concerning manic symptomatology, a difference was found in YMRS total scores between MEQ-E and MEQ-I (*p* < 0.001) as well as MEQ-E and MEQ-M (*p* = 0.006) (Table 4).

Given that, we performed a mediation analysis which is a statistical method used to quantify the causal sequence by which an antecedent variable causes a mediating variable that causes a dependent variable. Although mediation analysis is helpful for observational studies, and well fits the study design of the present research. The mediation analysis was performed to assess the mediation effect of PTH between MEQ-E and the psychopathological burden, as measured by the total score of HAM-A, HAM-D, and YMRS. As shown in Table 5, high PTH levels are directly affected by elated scores to HAM-A and HAM-D, and although the result is not statistically significant, we can also identify a trend for YMRS.

## 4. Discussion

To the best of our knowledge, this is the first study aimed at investigating the association between calcium imbalance and chronotype in BD patients. Our findings demonstrated a strong association between calcium imbalance and MEQ-E subjects. The mediation analysis highlighted a direct effect of high PTH levels in increasing the global psychopathological burden of BD symptomatology, as measured by HAM-D, HAM-A, and YMRS in MEQ-E individuals. As shown in our previous study, calcium metabolism directly affects the clinical course of BD, and it is correlated to different worsened clinical features and a higher psychopathological burden in BD [19]. The present results align with literature confirming that MEQ-E predisposes to the worst outcome. In addition, they highlight the association between calcium imbalance, MEQ-E, and the worst clinical outcome in BD compared to the other chronotypes [33]. In particular, our results highlight that most individuals with an eveningness chronotype (MEQ-E) are affected by BD-I (93.8%), as confirmed by some research [34,35]. However, this point is still highly debated in the literature since, according to a recent systematic review, when considering the BD types, data about rhythm comparisons between BD-I and BD-II is scarce and shows unclear results [25]. Therefore, this association between chronotype and clinical features in mood disorder has been widely discussed, and several studies suggested regarding MEQ-E as a predictor of poor prognosis [36], but never has the effect of calcium metabolism on this chronotype been explored before. Several investigations focused on the gene variant that can express the different circadian phenotypes, but only a few pointed out peripheral markers helpful in clinical practice. Indeed, despite the great genetic complexity underlying BD, there is a growing body of literature shining light on several genetic polymorphisms and genes potentially associated with circadian rhythm dysregulation, thus providing new perspectives on BD chronotypes [37]. In particular, future studies should investigate the most promising genes potentially able to explain the chronotype differences in the BD, including but not limited to CLOCK genes, acetylserotonin O-methyltransferase (ASMT) genes, and timeless (TIM) genes [25,38]. Evidence showed that a circadian clock operates in the parathyroid glands, and this downstream clock may affect the outcome of BD and part of the evening chronotype [39]. Based on this, it can be assumed there is a clear correlation between calcium imbalance and chronotype that directly influence the course of the BD.

Furthermore, several studies have suggested that MEQ-E may be a risk factor for depression due to a predisposition to a shortage of sleep caused by the circadian misalignment between biological and social time [40]. Indeed, insufficient sleep could lead to the habit of hiring antidepressant medications or tranquilizers in higher doses and often without medical advice [41]. This observation could explain our result in which antidepressant switch was higher in the MEQ-E group. Moreover, MEQ-E was associated with poor treatment response and a higher number of total episodes. During BD, eveningness is a marker of diminished response to first-line pharmacotherapy, such as lithium, and a predictor of increased severity of mood symptoms. This result aligns with our previous results that show a significant difference among the three chronotypes in psychopathological burden.

Additionally, several severe cognitive impairments are more related to MEQ-E patients than those with a morning preference. This could contribute to the explanation of the higher rate of psychotic characteristics in this population, as confirmed by our study [42]. Indeed, lithium-responsive patients tend to belong to the morning phenotype [43], while those under antidepressant therapy, such as selective serotonin reuptake inhibitors, tend to be part of the MEQ-E. In addition, evening-type patients tend to have higher bipolar disorder spectrum indices, including mood fluctuation mixed and anxious features, than those with early or intermediate orientations [44]. Another critical issue arising from our findings is the sticky correlation between the history of suicide and MEQ-E. This could be due to a significant psychopathological burden and the poor response to lithium treatment, as shown by studies on the argument.

Considering all the above, it follows that investigating the possible role of the chronotype in the phenotypic expression of bipolar disease and the different factors that can influence the course and its clinical outcome is of significant interest.

What emerged from our mediation analysis is that calcium metabolism, particularly PTH levels, can further deteriorate BD prognosis. In line with previous studies on the argument, we conducted the mediation analysis dividing chronotype in MEQ-E and not MEQ-E because much literature highlights no differences between morningness and intermediate chronotype. In light of the above, investigating the chronotype in BD and the different factors that can promptly mediate a worsening clinical outcome can be fundamental. The mediation analysis shows that calcium metabolism, particularly PTH levels, can further deteriorate BD prognosis. Mediation analysis was performed dividing chronotype in MEQ-E and not MEQ-E because much literature highlights no differences between morningness and intermediate chronotype. The PTH has a mediation effect in worsening all total scores psychometric tools. However, the calcium levels in the present study remained within the normal range despite PTH concentrations being higher in patients with a more significant psychopathological burden, supporting the hypothesis that PTH is a more reliable outcome measure than calcium imbalance since external factors can influence PTH in only a limited way, keeping it stable over time. A nuanced and complex molecular machinery can account for the self-regulation of CNS calcium signaling and the biochemical processes that influence circadian rhythms [45]. PTH secretion exhibits a diurnal variation with acrophase during the idle period in humans. The parathyroid glands are not in control of a superior “hypothalamic-pituitary-axis” as occurs for many endocrine glands, strengthening the idea of an autologous cycle regulation that can tune calcium signaling, not only in peripheral tissues but also in CNS, in the same way as with circadian rhythms [46]. Moreover, in humans, parathyroid glands present many CLOCK genes, which exhibit a diurnal variation [47]. Parathyroid hormone gene promoter contains an E-box-like element, a known target of circadian clock proteins expressed in several tissues. Circadian CLOCK genes are rhythmically expressed in normal parathyroid glands [48]. The E-box motifs are known targets of the central circadian clock proteins BMAL1 (Brain and Muscle ARNTL-like 1) and CLOCK genes [49]. Thus, molecular clock mechanisms exert a systemic functional regulation on hormonal secretion, metabolism, and cell cycle. Therefore, core clock genes and their products are involved in transcriptional-translational feedback loops and act as essential regulators of organ function [50]. The diurnal regulation of parathyroid glands was significantly associated with such rhythmicity [51]. Furthermore, the circadian clock operates in parathyroid glands, and this clock and downstream cell cycle regulators are widely distributed in CNS and peripheral tissues [52]. Notably, studies highlighted the role of fibroblast-growth-factor-receptor-1, MafB, and Gata3, commonly expressed in the PTH gland, and are involved in neuroinflammatory responses that affect the permeability of the Blood-Brain Barrier [47]. Prolonged neuroinflammation due to MEQ-E dis-rhythmicity lead to the dysregulated proliferation of parathyroid cells in secondary hyperparathyroidism. This mechanism could be responsible for calcium imbalance and lead to self-reinforcement of the neuroinflammation that in turn impairs mood symptoms. In addition to extracellular Ca^++^, the parathyroid gland is responsive to changes in plasma phosphate (pP) concentrations [53] cofactor FGFR1. The latter has recently been shown to be regulated by multiple factors such as the effect of P on CaSR, the activation of the vitamin D receptor, the fibroblast growth factor 23 (FGF23) through the growth factor receptor 1 (FGFR1) on the fibroblast, in combination with the Klotho, known for its properties related to the aging process, neurodegeneration, premature morbidity, and mortality [54]. Additionally, there is a linear relationship between circadian rhythm and calcium metabolism, especially with vitamin D considered a chrono-hormone and a regulator of parathyroid functioning [55]. In this regard, our hypothesis confirmed that vitamin D values were out of range in the MEQ-E subject. The direct evidence of a causal role of alterations in vitamin D levels and its functions in inducing depressive states is still debated. However, numerous neuronal processes correlate with the regulation of behaviors, suggesting its association with affective disorders; however, the specific pathogenic link of this correlation remains to be fully understood. In support of this notion, it must be kept in mind that VDR and vitamin D are widely detected in several areas relevant to depression in the human brain [56]. However, there is evidence that vitamin D antagonizes oxidative stress, blunts inflammation, favors neuroprotection, and modulates the homeostatic functions of various neuronal and glial cells, hence, supporting the hypothesis of its implication in the pathogenesis of affective disorders [57]. Although the exact molecular mechanisms linking vitamin D and depression remain not wholly investigated, one could argue that an imbalance in the calcium homeostasis of intracellular and extracellular compartments may lead to a significant potential fallout of disequilibrium between glutamate (an excitatory neurotransmitter) [58] and GABA (an inhibitory neurotransmitter) [59]. This mechanism, which in turn impairs neuronal functioning, affects cellular signaling. In addition, vitamin D has been reported to promote the synthesis of 5-HT, which plays an essential role in regulating mood, by enhancing the brain’s transcription of the serotonin-synthesizing gene tryptophan hydroxylase 2 (TPH2) [60].

Moreover, physiological vitamin D concentrations could also contribute to the fine-tuning of serotonin transmission into the brain by repressing monoaminoxidase-A and serotonin reuptake transporter (SERT). Furthermore, vitamin D displays neuroprotective effects and inhibits the synthesis of inflammatory cytokines [61], while previous evidence has proven the antioxidant properties of vitamin D, indicating that vitamin D lowers oxidative stress biomarkers [62]. The influence of the active form of vitamin D on the nervous system is associated with regulating the synthesis and release of neurotrophic factors. Some of them, such as nerve growth factor (NGF), and increased levels of glial cell line-derived neurotrophic factor (GDNF) [63], are considered to be involved in the pathogenesis of numerous neuropsychiatric diseases [64]. Vitamin D by rebalancing neurotrophin levels and rebalancing neurotransmitter alterations by regulating intracellular calcium stores and cellular signaling could improve mood disorders [65].

In addition, clinical trials indicate that vitamin D supplementation can improve depressive and anxiety symptomatology in BD due to its ability to restore alterations in neurotransmitters and neurotrophin levels in addition to its anti-inflammatory properties [66].

To conclude, our study is in line with what is reported by Nanou et al., according to which the correlation between bipolar symptoms and the imbalance of the calcium metabolism depends on a load of genetic alterations involving calcium signaling, which adversely impact synaptic functioning by altering the physiological transmission dynamics and the plasticity processes [67].

Despite the abovementioned significant preliminary findings, our study has several limitations that should be adequately discussed. Firstly, the naturalistic and cross-sectional nature of the study did not allow for the monitoring of PTH levels and vitamin D levels over time, or consider the type of psychopharmacological treatment. Secondly, since, according to exclusion criteria, those subjects with a concomitant drug able to alter calcium metabolism, such as lithium salt, the gold standard in treating BD, were not enrolled, one could argue that our findings could not be entirely generalized for the BD population. A further study could evaluate whether the eveningness chronotype could differently influence the effect of lithium on calcium metabolism or, rather, if an altered calcium metabolism could be influenced in lithium-treated BD subjects only if an eveningness chronotype is present. Furthermore, the prescription of sedative drugs such as benzodiazepines or antipsychotic medications should be considered or at least recognized as a limitation when evaluating chronotypes, although the enrollment of patients affected by BD-I in the absence of drug therapy appears far from everyday clinical practice. Thirdly, there is no control group and/or a comparator group (e.g., subjects with major depressive disorder [MDD]). One could argue that it would be interesting to deepen the role of PTH by stratifying BD patients according to the severity of illness and patients with mood disorders (both BD and MDD) according to the type of polarity or predominant illness course. Finally, the present study was first performed as a pilot exploratory study with a small sample size. A larger sample size could help clinicians better understand and potentially generalize the present findings.

In conclusion, the results of our pilot study suggest that MEQ-E may be a bipolarity-related marker and could predispose to different factors that exacerbate mood symptomatology. Although the mechanisms that link MEQ-E to a worse outcome in bipolarity could be mediated by different factors, investigating peripheral biomarkers, especially calcium metabolism, can help clinicians manage such a complex syndrome. Furthermore, the influence of the bipolarity-related aspect of MEQ-E must be distinct from poor sleep quality or lack of need to sleep and assessed as an independent clinical feature to prevent a worse clinical outcome.

Further analysis must investigate a preference for eveningness chronotype, and a high-amplitude circadian rhythm may be a potential diagnostic, prognostic, and therapeutic predictor. Nonetheless, implementing vitamin D therapy in MEQ-E can help improve the prognosis in this subtype of BD patients. A conclusion that we can deduce from our data is that implementing vitamin D therapy in MEQ-E can improve the prognosis in this subtype of BD patients.

## Figures and Tables

**Table 1:** Socio-demographic and clinical variables across different chronotypes: descriptive analyses.

Demographic Variables	MEQ-E (*n* = 32)	MEQ-I (*n* = 41)	MEQ-M (*n* =27)	*p*
Marital status, yes N (%)	12 (37.5)	22 (53.7)	12 (44.4)	0.638
Females, yes N (%)	17 (53.1)	15 (36.6)	18 (66.7)	0.058
Diploma, yes N (%)	24 (75.0)	32 (78.0)	21 (77.8)	0.951
Employed, yes N (%)	19 (59.4)	29 (70.7)	14 (51.9)	0.084
Family Psychiatric History, yes N (%)	25 (78.1)	25 (61.0)	18 (66.7)	0.276
Bipolar type 1, yes N (%)	30 (93.8)	15 (36.6)	10 (37.0)	**<0.001**
Age, M (SD)	46.8 (15.5)	46.3 (11.4)	46.3 (15.9)	0.978

M: mean; MEQ-E: Eveningness chronotype; MEQ-I: Intermediate Chronotype; MEQ-M: Morningness Chronotype; N: total number; SD: standard deviation; %: percentage. Bold *p*-values indicate a significant level.

**Table 2:** Psychopathological features across different chronotypes: χ^2^ test.

Psychopathological Features Comparison among the Different Chronotypes						
	MEQ-E	MEQ-I	MEQ-M	χ^2^	df	*p*
Antidepressant switch, N (%)	19 (65.5%)	4 (13.8%)	6 (20.7%)	22.316	2	**<0.001**
Aggressive behavior, N (%)	24 (42.1%)	21 (36.8%)	12 (21.1%)	6.526	2	**0.038**
Mixed features, N (%)	30 (62.5%)	10 (20.8%)	8 (16.7%)	38.926	2	**<0.001**
Anxious features, N (%)	30 (62.5%)	19 (30.2%)	14 (22.2%)	19.301	2	**<0.001**
Psychotic symptoms, N (%)	30 (68.2%)	9 (20.5%)	5 (11.4%)	47.348	2	**<0.001**
History of Suicide, N (%)	21 (65.6%)	5 (15.6%)	6 (18.8%)	25.204	2	**<0.001**
	**MEQ-E^+^**	**MEQ-E^-^**		**χ^2^**	**df**	***p***
Antidepressant switch, N (%)	19 (65.5%)	10 (34.5%)		21.087	1	**<0.001**
Aggressive behavior, N (%)	24 (42.1%)	33 (57.9%)		6.221	1	**0.017**
Mixed features, N (%)	30 (62.5%)	18 (37.5%)		38.788	1	**<0.001**
Anxious features, N (%)	30 (47.6%)	33 (52.4%)		19.089	1	**<0.001**
Psychotic symptoms, N (%)	30 (68.2%)	14 (31.8%)		47.270	1	**<0.001**
History of Suicide, N (%)	21 (65.6%)	11 (34.4%)		24.452	1	**<0.001**

MEQ-E: Eveningness chronotype; MEQ-E^-^: Intermediate and Morningness Chronotypes; MEQ-I: Intermediate Chronotype; MEQ-M: Morningness Chronotype; N: total number; %: percentage. Bold *p*-values indicate a significant level.

**Table 3:** Clinical features across different chronotypes: Kruskal-Wallis test.

	MEQ-E	MEQ-I	MEQ-M	χ^2^	df	*p*	ε²
Number of total episodes, M (SD)	17.7 (12.8)	7.9 (6.1)	5.9 (3.7)	23.337	2	**<0.001**	0.23573
Number of depressive episodes, M (SD)	9.0 (6.6)	4.1 (3.2)	3.1 (1.9)	20.309	2	**<0.001**	0.20724
Number of manic episodes, M (SD)	4.6 (3.2)	3.3 (2.5)	2.4 (1.5)	5.168	2	0.075	0.09570
Number of hypomanic episodes, M (SD)	4.8 (4.6)	2.6 (1.6)	2.2 (1.0)	6.183	2	**0.045**	0.06648
Age at first psychiatric contact, M (SD)	29.8 (8.5)	29.6 (9.5)	30.6 (12.7)	0.242	2	0.886	0.00244
Age at illness onset, M (SD)	27.1 (7.9)	26.6 (9.1)	28.7 (12.9)	0.669	2	0.716	0.00676
HAM-D, M (SD)	46.4 (11.2)	27.1 (11.8)	23.9 (11.6)	43.330	2	<0.001 *	0.4377
HAM-A, M (SD)	21.1 (8.3)	7.9 (9.5)	8.5 (9.6)	28.648	2	<0.001 *	0.2894
YMRS, M (SD)	29.1 (6.4)	18.7 (8.7)	21.3 (9.9)	22.293	2	<0.001 *	0.2252
Calcium levels, M (SD)	9.5 (0.5)	9.4 (±0.4)	9.5 (0.4)	1.706	2	0.426 *	0.0172
PTH levels, M (SD)	62.2 (12.6)	40.8 (17.6)	38.5 (21.7)	27.114	2	<0.001 *	0.2739
Vitamin D levels, M (SD)	33.3 (11.2)	44.2 (47.6)	38.9 (10.9)	6.069	2	0.048 *	0.0613

HAM-A: Hamilton Anxiety Rating Scale; HAM-D: Hamilton Depression Rating Scale; M: mean; MEQ-E: Eveningness chronotype; MEQ-I: Intermediate Chronotype; MEQ-M: Morningness Chronotype; N: total number; PTH: Parathormon; SD: standard deviation; YMRS: Young Mania Rating Scale. %: percentage. * Fisher’s Exact test. Bold *p*-values indicate a significant level.

**Table 4:** Dwass-Steel-Critchlow-Fligner pairwise comparisons.

**Dwass-Steel-Critchlow-Fligner Pairwise Comparisons for Psychopathological Scale**							
**Chronotype**		**HAMD Total Score**		**HAMA Total Score**		**YMRS Total Score**	
		w	*p*	w	*p*	w	*p*
MEQ-E	MEQ-I	−8.112	**<0.001**	−6.946	**<0.001**	−6.61	**<0.001**
MEQ-E	MEQ-M	−8.034	**<0.001**	−5.900	**<0.001**	−4.34	**0.006**
MEQ-I	MEQ-M	−0.532	0.925	0.834	0.826	1.43	0.571
**Dwass-Steel-Critchlow-Fligner Pairwise Comparisons for Biological Variable**							
**Chronotype**		**Ca^++^ Levels**		**PTH Levels**		**Vitamin D Levels**	
		w	*p*	w	*p*	w	*p*
MEQ-E	MEQ-I	−1.510	0.534	−6.71	**<0.001**	2.43	0.198
MEQ-E	MEQ-M	−0.716	0.868	−5.87	**<0.001**	3.35	**0.047**
MEQ-I	MEQ-M	1.531	0.525	−1.38	0.591	1.30	0.627

MEQ-E: Eveningness chronotype; MEQ-I: Intermediate Chronotype; MEQ-M: Morningness Chronotype. Bold *p*-values indicate a significant level.

**Table 5:** (a) Mediation Analyses. (b) Mediation HAM-D. (c) Mediation HAM-A.

Mediation Analyses										
a. Mediation YMRS										
						**95% Confidence Interval**				
Effect			**Label**	**Estimate**	**SE**	**Lower**	**Upper**	**Z**	***p***	**% Mediation**
Indirect			a × b	−1.08	1.09	−3.2	1.13	−0.997	0.319	11.6
Direct			c	−8.24	1.66	−11.45	−4.79	−4.955	**<0.001**	88.4
Total			c + a × b	−9.33	1.56	−12.45	−6.34	−5.968	**<0.001**	100
Path Estimates										
						**95% Confidence Interval**				
			**Label**	**Estimate**	**SE**	**Lower**	**Upper**	**Z**	***p***	
MEQ-E	→	PTH	a	−22.3409	3.1036	−28.4843	−16.339	−7.2	**<0.001**	
PTH	→	YMRS	b	0.0485	0.0482	−0.0466	0.14	1	0.315	
MEQ-E	→	YMRS	c	−8.2447	1.6639	−11.4546	−4.789	−4.96	**<0.001**	
b. Mediation HAM-D.										
						**95% Confidence Interval**				
Effect			**Label**	**Estimate**	**SE**	**Lower**	**Upper**	**Z**	***p***	**% Mediation**
Indirect			a × b	−5.15	1.75	−9.03	−2.24	−2.94	**0.003**	25.5
Direct			c	−15.05	2.86	−20.51	−8.95	−5.26	**<0.001**	74.5
Total			c + a × b	−20.2	2.39	−24.77	−15.4	−8.45	**<0.001**	100
Path Estimates										
						**95% Confidence Interval**				
			**Label**	**Estimate**	**SE**	**Lower**	**Upper**	**Z**	***p***	
MEQ-E	→	PTH	a	−22.341	3.0735	−27.964	−15.984	−7.27	**<0.001**	
PTH	→	HAM-D	b	0.231	0.0656	0.108	0.371	3.51	**<0.001**	
**MEQ-E**	→	HAM-D	c	−15.046	2.8577	−20.508	−8.946	−5.26	**<0.001**	
c. Mediation HAM-A.										
						**95% Confidence Interval**				
Effect			**Label**	**Estimate**	**SE**	**Lower**	**Upper**	**Z**	***p***	**% Mediation**
Indirect			a × b	−3.16	1.24	−5.89	−1.14	−2.56	**0.01**	27.5
Direct			c	−8.32	2.05	−12.13	−4.18	−4.07	**<0.001**	72.5
Total			c + a × b	−11.48	1.72	−14.85	−8.15	−6.66	**<0.001**	100
Path Estimates										
						**95% Confidence Interval**				
			**Label**	**Estimate**	**SE**	**Lower**	**Upper**	**Z**	***p***	
MEQ-E	→	PTH	a	−22.341	3.3179	−28.6972	−15.307	−6.73	**<0.001**	
PTH	→	HAM-A	b	0.142	0.0477	0.0518	0.243	2.97	**0.003**	
MEQ-E	→	HAM-A	c	−8.318	2.0458	−12.1337	−4.181	−4.07	**<0.001**	

HAM-A: Hamilton Anxiety Rating Scale; HAM-D: Hamilton Depression Rating Scale; MEQ-E: Eveningness chronotype; PTH: Parathormon; YMRS: Young Mania Rating Scale. →: Mediation effect. Bold *p*-values indicate a significant level.

## Data Availability

The data presented in this study are available on request from the corresponding author. The data are not publicly available due to privacy issues.
